# Human cytomegalovirus UL141 promotes efficient downregulation of the natural killer cell activating ligand CD112

**DOI:** 10.1099/vir.0.021931-0

**Published:** 2010-08

**Authors:** Virginie Prod'homme, Daniel M. Sugrue, Richard J. Stanton, Akio Nomoto, James Davies, Carole R. Rickards, Daniel Cochrane, Melanie Moore, Gavin W. G. Wilkinson, Peter Tomasec

**Affiliations:** 1 Department of Infection, Immunity and Biochemistry, Section of Medical Microbiology, School of Medicine, Cardiff University, Cardiff, UK; 2 Department of Microbiology, Graduate School of Medicine, University of Tokyo, Japan

## Abstract

Human cytomegalovirus (HCMV) UL141 induces protection against natural killer cell-mediated cytolysis by downregulating cell surface expression of CD155 (nectin-like molecule 5; poliovirus receptor), a ligand for the activating receptor DNAM-1 (CD226). However, DNAM-1 is also recognized to bind a second ligand, CD112 (nectin-2). We now show that HCMV targets CD112 for proteasome-mediated degradation by 48 h post-infection, thus removing both activating ligands for DNAM-1 from the cell surface during productive infection. Significantly, cell surface expression of both CD112 and CD155 was restored when UL141 was deleted from the HCMV genome. While gpUL141 alone is sufficient to mediate retention of CD155 in the endoplasmic reticulum, UL141 requires assistance from additional HCMV-encoded functions to suppress expression of CD112.

Human cytomegalovirus (HCMV), the prototype species of the subfamily *Betaherpesvirinae*, has a high prevalence in populations worldwide. Although HCMV is recognized to be an important human pathogen, particularly in immunocompromised individuals or following congenital infection, the vast majority of primary infections are subclinical and accompanied by asymptomatic lifelong carriage. HCMV encodes highly effective systems to provide for latency, persistent reactivation and transmission; as part of this process the virus acquired an impressive array of genes that act both to evade and redirect the host immune response (Wilkinson *et al.*, 2008). The fact that individuals with genetic defects in their natural killer (NK) cell response are particularly susceptible to severe HCMV disease (Biron *et al.*, 1989; Gazit *et al.*, 2004) provided a rationale to focus attention on this arm of the immune response.

NK cells are composed of heterogeneous populations expressing a ‘mosaic’ of different activating and inhibitory receptors, the function of each cell being regulated by integration of signals received from ligands presented on potential target cells (Lanier, 2008). Inhibitory signals received mainly from autologous MHC class-I molecules normally dominate, to maintain NK cells in a resting state. However, HCMV not only efficiently downregulates MHC-I (Ahn *et al.*, 1997; Furman *et al.*, 2002; Jones *et al.*, 1996; Trgovcich *et al.*, 2006; Wiertz *et al.*, 1996a, b), but also stimulates the expression of recognized NK cell activating ligands, e.g. MHC-I-related chains (MIC) A and B, UL16-binding proteins (ULBP) 1–3, retinoic acid early transcripts (RAET)1E/ULBP4, RAET1G/ULBP5, RAET1L/ULBP6 and CD155 (Bacon *et al.*, 2004; Bahram *et al.*, 1994; Bauer *et al.*, 1999; Chalupny *et al.*, 2003; Cosman *et al.*, 2001; Eagle *et al.*, 2009; Groh *et al.*, 2001; Tomasec *et al.*, 2005). Despite this, HCMV-infected cells actually prove to be highly resistant to NK cells in functional assays (Cerboni *et al.*, 2000; Tomasec *et al.*, 2005). This resilience can be attributed to a substantial proportion of HCMV genome being directed towards evading the NK cell response.

Although HCMV downregulates endogenous MHC-I, the virus also encodes its own MHC-I homologue (gpUL18) that binds the inhibitory receptor LIR-1 (ILT-2) with high affinity (Beck & Barrell, 1988; Chapman *et al.*, 1999; Prod'homme *et al.*, 2007) and a peptide in the UL40 leader sequence that acts to promote cell surface expression of the non-classical MHC-I molecule HLA-E, the ligand for the inhibitory receptor CD94/NKG2A (Tomasec *et al.*, 2000; Ulbrecht *et al.*, 2000; Wang *et al.*, 2002). The activating receptor NKG2D is remarkable in recognizing eight ligands. To combat their activation UL16 retains MICB, ULBP1 and ULBP2 in the endoplasmic reticulum (ER); miR-UL112 targets the MICB transcript, while UL142 downregulates MICA (Chalupny *et al.*, 2006; Cosman *et al.*, 2001; Stern-Ginossar *et al.*, 2007; Wills *et al.*, 2005). The NK cell activating receptor DNAM-1 (CD226) recognizes both CD155 and CD112 (Bottino *et al.*, 2003; Fuchs *et al.*, 2004). We previously demonstrated that UL141 elicits efficient protection against NK cell-mediated cytolysis by sequestering CD155 in the ER yet, in isolation, had no effect on CD112 (Tomasec *et al.*, 2005).

CD155 is the poliovirus receptor (PVR) or nectin-like molecule-5 (necl-5), while CD112 is also referred to as nectin-2, herpesvirus entry mediator B (HVEB) or poliovirus receptor-related protein 2 (PRR2). CD112 and CD155 are both structurally and functionally related. Nectins and necls are immunoglobulin-like molecules involved in cell adhesion, movement, proliferation, differentiation, polarization, virus entry and immune recognition (Takai *et al.*, 2008). In view of its important role as an activating ligand for DNAM-1, we sought to analyse CD112 expression in the context of HCMV infection. Initial flow cytometry studies revealed that CD112 was downregulated by the low passage HCMV strain Merlin, but not high passage strain AD169 (not shown). Strain AD169 has a 15 kb deletion encompassing UL132–UL150 that includes the NK cell evasion genes UL141 and UL142. Merlin was derived from a bacterial artificial chromosome (BAC) containing the entire strain Merlin genome (R. J. Stanton, unpublished data). MerlinΔUL141 was generated using technologies developed previously to facilitate manipulation of the adenovirus genome (Stanton *et al.*, 2008). Briefly, a selectable cassette comprising ampicillin resistance, lacZ and SacB was PCR amplified and recombineered into the Merlin BAC in place of nt 184597–185412 (relative to published Merlin sequence GenBank accession no. NC_006273) using primers SacBF-UL141 (5′-caggtagcataggaaacatacggtgaaaatactccaaaatcccaaaaatgccgcgattccccgagtggcccagggagacctgtgacggaagatcacttcg-3′, homology to pAL1111 underlined) and SacBR-UL141 (5′-ccgacgtttgagcggccgacacacggagcaggaacaggcgggcagcgtctctgcgaaaaagggaagaaaagaatcatcctgaggttcttatggctcttg-3′, homology to pAL1111 underlined). In a second recombineering step, the selectable cassette was removed using oligo delUL141 (5′-atactccaaaatcccaaaaatgccgcgattccccgagtggcccagggagagatgattcttttcttccctttttcgcagagacgctgcccgcctgttcctg-3′), leaving behind a seamless deletion of the first 816 bp of the UL141 ORF.

In human fetal foreskin fibroblasts (HFFF) infected with Merlin, cell surface levels of CD155, CD112 and MHC-I were progressively downregulated over the course of infection (Fig. 1), with the change in CD112 being more pronounced at 48 h post-infection (p.i.) (Fig. 1b). In accord with previous observations (Tomasec *et al.*, 2005), cells infected with MerlinΔUL141 had elevated cell surface levels of CD155, while CD112 levels were comparable with the mock-infected HFFF (Fig. 1). Deletion of UL141 therefore ablated downregulation of both CD155 and CD112. This restoration of CD112 expression was unexpected, since UL141 had no overt effect on CD112 when expressed in isolation (Tomasec *et al.*, 2005). Interestingly, a small reproducible decrease in CD112 persisted when MerlinΔUL141-infected and mock-infected cells were compared at 96 h p.i. (Fig. 1d). Replicate samples from the flow cytometry study were analysed by immunoblot, in order to further assess the fate of the CD112 protein within the cell. Briefly, cells were extracted with Triton X-114 (Bordier, 1981), proteins were separated on NuPAGE gels (Invitrogen) and blots were analysed with two independent polyclonal anti-CD112 antibodies. In Merlin-infected cells, the loss of CD155 from the cell surface (Fig. 1) correlated with the emergence of elevated levels of an immature (endoglycosidase H-sensitive) form of CD155 complexed with gpUL141 in the ER (Cochrane, 2009; Tomasec *et al.*, 2005) (Fig. 2a). In contrast to CD155, the CD112 signal gradually decreased in Merlin-infected cells and was not detected by 72 h p.i. (Fig. 2a).

Quantitative real time-PCR showed CD112 mRNA levels to be marginally increased throughout the infection (not shown), consistent with CD112 expression being regulated post-transcriptionally. To determine whether CD112 was targeted for proteolytic degradation, Merlin-infected cells were incubated in the presence of proteasome inhibitors. Treatment with either MG132 or Epoxomycin (Calbiochem) was able to restore CD112 expression, indicating that HCMV targeted CD112 for proteasome-mediated degradation (Fig. 2b).

UL141 was required for efficient downregulation of both CD112 and CD155 from the cell surface in HCMV-infected cells (Figs 1 and 3a), yet had no effect on CD112 in cells infected with recombinant adenovirus vector encoding UL141 [RAdUL141 (Tomasec *et al.*, 2005); Fig. 3b]. We reasoned that UL141 acted in partnership with an additional HCMV-encoded function(s) to downregulate CD112. Indeed, the residual level of CD112 suppression mediated by the MerlinΔUL141 (Figs 1d, 2a and 3a) could potentially be mediated by this function operating suboptimally. In cells co-infected with MerlinΔUL141 and RAdUL141, the HCMV deletion mutant was complemented; downregulation of both CD112 and CD155 was restored (Fig. 3c). Similarly, co-infection of strain AD169 with RAdUL141 also resulted in the downregulation of both CD112 and CD155 (Fig. 3d). These data are consistent with UL141 co-operating with additional HCMV-expressed function(s) to efficiently downregulate CD112, and that function also being intact within AD169 strain (thus excluding UL133–150). Through downregulation of CD112, HCMV eliminates from the cell surface an activating ligand for DNAM-1, which presumably contributes to the enhanced killing of HCMV-infected cells observed when UL141 is deleted from the virus (Fig. 3e, f), but not to the protection elicited when UL141 is expressed in isolation (Tomasec *et al.*, 2005). HCMV thus targets both ligands for the NK cell activating receptor DNAM-1. GpUL141 alone is sufficient to sequester CD155 in the ER, while this study predicts that gpUL141 acts in concert with an additional viral function to induce proteasome-mediated degradation of CD112. This additional viral function could either directly co-operate with UL141, or act upon a cellular intermediate.

DNAM-1 is remarkable in being expressed on all NK cells and plays a major role in regulating their function. HCMV suppression of CD112 and CD155 may have ramifications that extend beyond the regulation of NK cell function. DNAM-1 is also expressed on activated T, NKT, myeloid and mast cells, megakaryocytes, platelets and a subset of B lymphocytes thereby impacting on a wide range of immunological responses and regulating platelet activation (Bachelet *et al.*, 2006; Bottino *et al.*, 2003; Burns *et al.*, 1985; Kojima *et al.*, 2003; Pende *et al.*, 2006; Reymond *et al.*, 2004; Scott *et al.*, 1989; Shibuya *et al.*, 1996, 1999, 2003; Xu & Jin, 2010). For example, the interaction between DNAM-1 and CD112/CD155 has been associated with T-cell differentiation, proliferation, cytotoxicity and cytokine secretion (Tahara-Hanaoka *et al.*, 2004). Furthermore, nectins and necls regulate fundamental processes in cell biology including cell adhesion, movement, proliferation, differentiation, survival, polarization and signalling (Takai *et al.*, 2008). HCMV infection is recognized to disrupt focal adhesions and intercellular connections, while inducing cell motility and transendothelial migration (Chan *et al.*, 2009; Stanton *et al.*, 2007). It will be important to determine how the modulation of CD112 and CD115 influences these processes.

## Figures and Tables

**Fig. 1.**
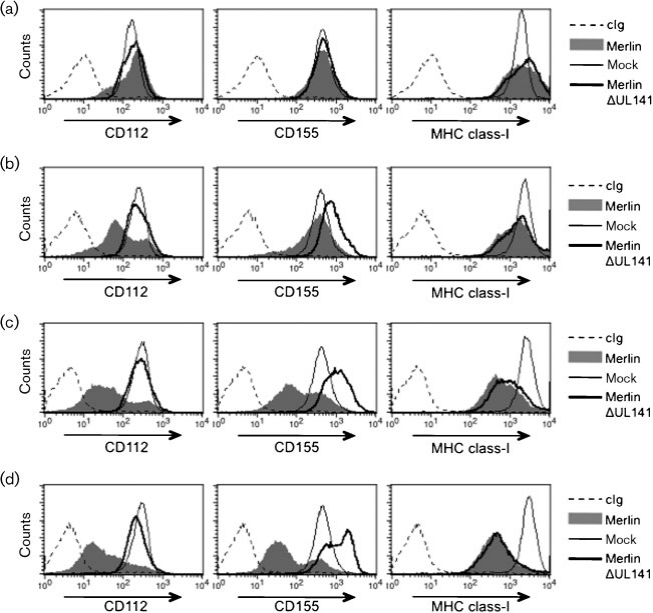
HFFF were infected (m.o.i.=25) for (a) 24 h (b) 48 h (c) 72 h or (d) 96 h with HCMV strain Merlin, MerlinΔUL141 or mock-infected and cell surface expression of CD112 (Santa Cruz, sc-65333) was analysed by flow cytometry. For reference, expression levels of CD155 (Abcam, ab-3142) and MHC class-I (W632; ATCC) were also monitored, alongside control Ig (cIg).

**Fig. 2.**
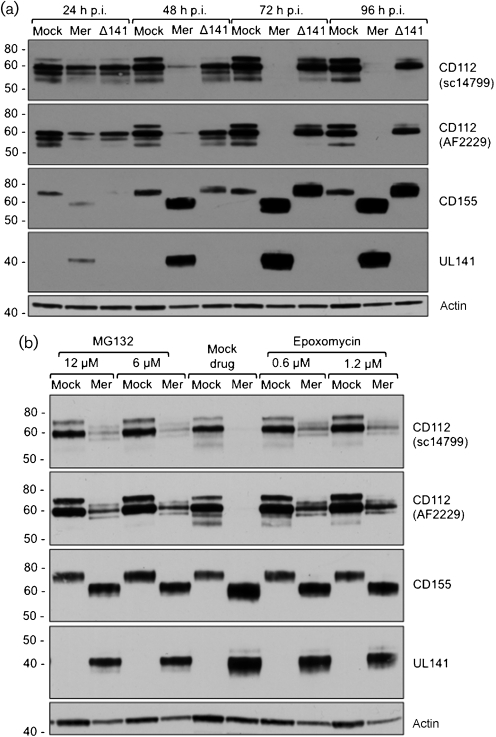
HFFF were infected (m.o.i.=25) for 24, 48, 72 or 96 h p.i. with HCMV strain Merlin (Mer), MerlinΔUL141 (Δ141) or mock-infected (Mock) and cell extracts were analysed by immunoblot using antibodies to: CD112 (R&D, AF2229; Santa Cruz, sc-14799), CD155 [5D1 (Aoki *et al.*, 1994)], UL141 [M550 (Tomasec *et al.*, 2005)] and actin (A-2066; Sigma). (b) HFFF were infected (m.o.i.=25) for 48 h with HCMV strain Merlin (Mer) or mock-infected, then treated for 12 h with proteasome inhibitors MG132 or Epoxomycin as indicated and analysed by immunoblot as in (a).

**Fig. 3.**
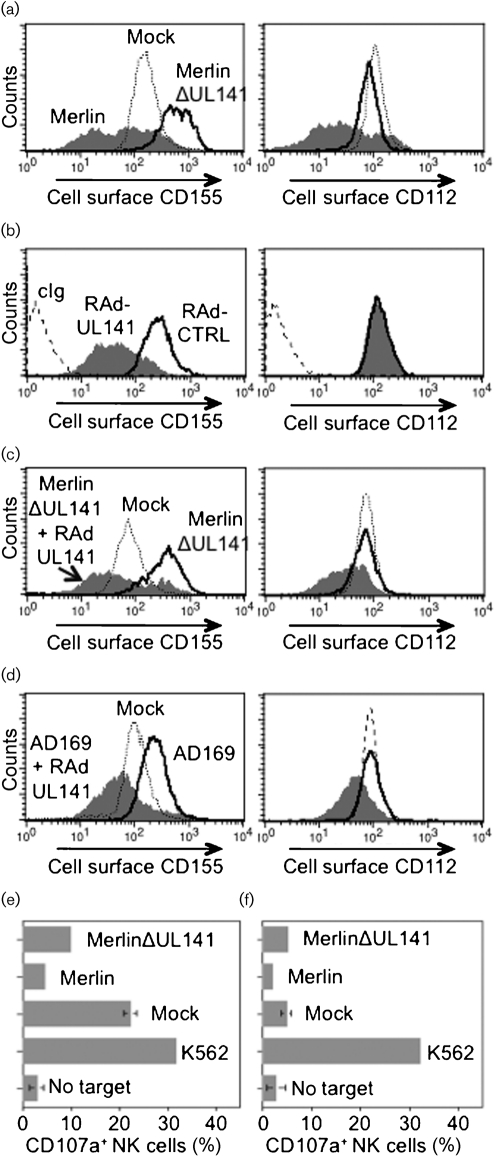
HFFF were infected for 72 h (m.o.i.=25) with HCMV strain Merlin or MerlinΔUL141, as indicated, and analysed for cell surface expression of CD155 and CD112 by flow cytometry. (b) HFFF were infected for 72 h (m.o.i.=200) with replication-deficient adenovirus vectors encoding HCMV UL141 (RAd-UL141) or equivalent empty RAd (RAd-CTRL) (Tomasec *et al.*, 2005), as indicated, and analysed for cell surface expression of CD155 and CD112 by flow cytometry. (c) HFFF were co-infected for 72 h with MerlinΔUL141+RAd-CTRL or MerlinΔUL141+RAd-UL141, as indicated, and analysed for cell surface expression of CD155 and CD112 by flow cytometry. (d) HFFF were co-infected for 72 h with HCMV strain AD169+RAd-CTRL (AD169) or AD169+RAd-UL141, as indicated, and analysed for cell surface expression of CD155 and CD112 by flow cytometry. Control Ig histograms (cIg) were not included in panels (a), (c) and (d) to maintain figure clarity. (e) HFFF were infected for 72 h with HCMV strain Merlin, MerlinΔUL141 or mock infected. Sensitivity to NK cells was measured using alpha interferon (IFN-*α*) activated PBMC in allogeneic CD107a mobilization assay (Prod'homme *et al.*, 2007) using the following antibodies: anti-CD107a-FITC (553793; BD Biosciences), anti-CD3-PerCP (SK7; BD Biosciences), anti-CD56-APC (N901; Beckman Coulter). PBMC incubated without targets and K562 cells are shown as controls. (f) RS primary skin fibroblasts were infected for 72 h with HCMV strain Merlin, MerlinΔUL141 or mock infected. Sensitivity to NK cells was measured using IFN-*α* activated RS PBMC in autologous CD107a mobilization assay as described in (e).
